# Translocation of Dense Granule Effectors across the Parasitophorous Vacuole Membrane in *Toxoplasma-*Infected Cells Requires the Activity of ROP17, a Rhoptry Protein Kinase

**DOI:** 10.1128/mSphere.00276-19

**Published:** 2019-07-31

**Authors:** Michael W. Panas, Abel Ferrel, Adit Naor, Elizabeth Tenborg, Hernan A. Lorenzi, John C. Boothroyd

**Affiliations:** a Department of Microbiology and Immunology, Stanford School of Medicine, Stanford, California, USA; b Department of Infectious Diseases, J. Craig Venter Institute, Rockville, Maryland, USA; c University of California at Davis, School of Veterinary Medicine, Davis, California, USA; Indiana University School of Medicine

**Keywords:** *Toxoplasma gondii*, effector proteins, host-parasite relationship, kinases

## Abstract

Toxoplasma gondii tachyzoites co-opt host cell functions through introduction of a large set of rhoptry- and dense granule-derived effector proteins. These effectors reach the host cytosol through different means: direct injection for rhoptry effectors and translocation across the parasitophorous vacuolar membrane (PVM) for dense granule (GRA) effectors. The machinery that translocates these GRA effectors has recently been partially elucidated, revealing three components, MYR1, MYR2, and MYR3. To determine whether other proteins might be involved, we returned to a library of mutants defective in GRA translocation and selected one with a partial defect, suggesting it might be in a gene encoding a new component of the machinery. Surprisingly, whole-genome sequencing revealed a missense mutation in a gene encoding a known rhoptry protein, a serine/threonine protein kinase known as ROP17. ROP17 resides on the host cytosol side of the PVM in infected cells and has previously been known for its activity in phosphorylating and thereby inactivating host immunity-related GTPases. Here, we show that null or catalytically dead mutants of ROP17 are defective in GRA translocation across the PVM but that translocation can be rescued “in *trans*” by ROP17 delivered by other tachyzoites infecting the same host cell. This strongly argues that ROP17’s role in regulating GRA translocation is carried out on the host cytosolic side of the PVM, not within the parasites or lumen of the parasitophorous vacuole. This represents an entirely new way in which the different secretory compartments of *Toxoplasma* tachyzoites collaborate to modulate the host-parasite interaction.

**IMPORTANCE** When *Toxoplasma* infects a cell, it establishes a protective parasitophorous vacuole surrounding it. While this vacuole provides protection, it also serves as a barrier to the export of parasite effector proteins that impact and take control of the host cell. Our discovery here that the parasite rhoptry protein ROP17 is necessary for export of these effector proteins provides a distinct, novel function for ROP17 apart from its known role in protecting the vacuole. This will enable future research into ways in which we can prevent the export of effector proteins, thereby preventing *Toxoplasma* from productively infecting its animal and human hosts.

## Abstract (precis)

When *Toxoplasma* infects a cell, it establishes a protective parasitophorous vacuole surrounding it. While this vacuole provides protection, it also serves as a barrier to the export of parasite effector proteins that impact and take control of the host cell. Our discovery here that the parasite rhoptry protein ROP17 is necessary for export of these effector proteins provides a distinct, novel function for ROP17 apart from its known role in protecting the vacuole. This will enable future research into ways in which we can prevent the export of effector proteins, thereby preventing *Toxoplasma* from productively infecting its animal and human hosts.

## INTRODUCTION

Toxoplasma gondii is an obligate intracellular parasite capable of infecting a wide range of cell types in almost any warm-blooded animal. As for most *Apicomplexa*, entry into a host cell and interaction with host functions once inside involve the coordinated action of at least three distinct secretory compartments: micronemes, rhoptries, and dense granules (1). The small, apical micronemes are the first to function in invasion, releasing adhesins onto the surface of the parasite that are crucial for attachment to the host cell (2, 3). The much larger, bulb-shaped rhoptries are also apically located, and these somehow directly introduce their contents into the host cell at the start of actual invasion (4). Once initiated, invasion involves an invagination of the host plasma membrane to form a parasitophorous vacuole (PV). This process is mediated by binding between a surface-localized micronemal protein, AMA1, and RON2, a protein that starts in the rhoptry necks (hence “RON”) but is introduced into the host cell to become an integral membrane protein within the host plasma membrane (5–7).

In addition to the RON proteins, rhoptries also introduce the contents of their bulbs during invasion (8, 9). These proteins, generally known as ROPs, are a set of effectors whose job generally appears to be to co-opt host functions (4, 10–12). Most known ROPs are members of an extended family of protein kinases and pseudokinases defined by their homology to the prototypical member of the family, ROP2 (13). Among the active ROP2-like kinases are ROP17 and ROP18 which have been well studied for their role in defense against immunity-related GTPases (IRGs), a set of host proteins that are generated in response to interferon gamma and that attack the parasitophorous vacuole membrane (PVM), resulting in eventual death of the parasites inside (14–17). In collaboration with the pseudokinase ROP5, ROP17 and ROP18 phosphorylate IRGs, which disrupts their ability to bind GTP, thereby neutralizing their ability to attack the PVM (18–21). The location of these ROPs at the PVM (22, 23), specifically on the host cytosolic side of this membrane (24), perfectly positions them for their role in defending against IRG attack. In addition, ROP17 has been shown to impact the host transcriptional network directly by as yet unknown means (25) and to be essential for full virulence in mice (21, 26).

A third secretory compartment, the dense granules, also plays a key function in the interaction with the host cell. The contents of these spherical organelles are known as GRAs, and they are released into the PV after invasion is under way (27). Unlike rhoptry proteins, however, GRAs are not injected directly into the host cytosol but instead are secreted into the PV space (28–30). Some GRA proteins are involved in elaboration of the PVM into a complex network of nanotubes known as the intravacuolar network (31, 32). Others associate with or even integrate into the PVM where they mediate a variety of host functions, including recruitment of host mitochondria (33), activation of host NF-κB (34), and activation of NFAT4 (35). A third set of GRA proteins, including GRA16, GRA18, GRA24, and TgIST, however, are translocated across the PVM and into the host cytosol, with some eventually reaching the host nucleus where they have a profound effect on many host functions (11, 36). This class of GRA proteins impacts the activity of host p53 (37) p38 mitogen-activated protein (MAP) kinase (38), STAT1 signaling (39, 40), beta-catenin signaling (41), E2F signaling (42), and c-Myc expression (43).

Using a genetic screen for *Toxoplasma* genes necessary for the aforementioned host c-Myc upregulation, we previously demonstrated that the translocation of GRAs across the PVM involves a set of parasite proteins that originate in dense granules or dense granule-like organelles, ultimately reaching the PVM (43, 44). These MYR (Myc regulation) genes were identified by using fluorescence-activated cell sorting (FACS) to select from a population of chemically mutagenized *Toxoplasma* tachyzoites those mutants that fail to upregulate a green fluorescent protein (GFP)−c-Myc reporter fusion in bone marrow macrophages. Whole-genome sequencing of clones from the resulting populations of *Toxoplasma* mutants revealed three novel genes as necessary for the c-Myc upregulation, *MYR1*, *MYR2*, and *MYR3* (43, 44). MYR1 and MYR3 form a stable complex at the PVM (44), and it is presumed that these two proteins are part of a translocon system that mediates the movement of GRAs across this membrane. MYR2 is also at the periphery of the PV, but it has not yet been found to associate with either of the other two MYR proteins. Deletion of any one of the three *MYR* genes results in a complete loss of GRA translocation, and as expected for a mutant that cannot introduce an entire class of crucial effector proteins, Δ*myr1* strains have a much-reduced impact on the host transcriptome (42); they are also substantially attenuated in a mouse model of virulence (43).

*A priori*, it seemed likely that more than just these three proteins would be necessary for the translocation of GRA effectors across the PVM. To address this possibility, we returned to the original library of Myr^−^ mutants (43) and asked whether any of the mutants had a partial defect which might indicate that they were defective in a gene other than *MYR1/2/3*. We report here the isolation of one such mutant which was found to have a missense mutation in *ROP17* and go on to show that a functional ROP17 within the host cell is indeed necessary for the translocation of GRA proteins across the PVM, indicating that a rhoptry-derived kinase located at the PVM plays an unanticipated role in this crucial process.

## RESULTS

**FIG 1:**
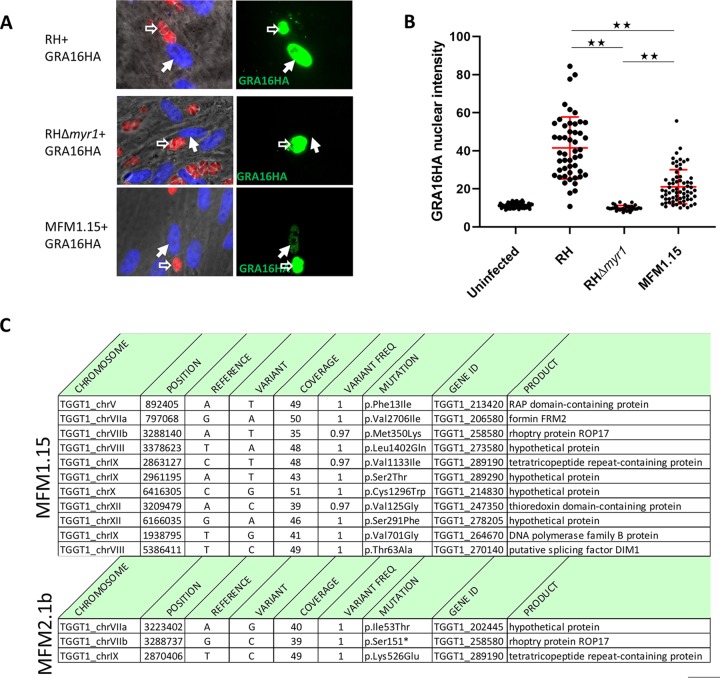
MFM1.15 shows a partial Myr^−^ phenotype and has a mutation in *ROP17*. (A) Representative immunofluorescence assay (IFA) images of HFFs infected with wild-type RH (RH-WT), RHΔ*myr1*, or RH mutant MFM1.15. All three parasite strains express cytosolic td-tomato (red) and were transfected with a plasmid expressing HA-tagged GRA16 which was detected by probing with anti-HA (green). Hollow white arrows indicate vacuoles containing parasites expressing the GRA16HA transgene. Solid white arrows indicate the nuclei in cells containing such vacuoles. Only the RH-WT-infected cells show efficient translocation of GRA16HA to the host cell nucleus. Images from one of six biological replicates are shown. (B) Quantitation of nuclear GRA16HA was assessed by anti-HA tag staining followed by ImageJ to determine the intensity of nuclear staining in infected cells by transiently transfected parasites from at least 10 random fields. The MFM1.15 mutant shows a significantly reduced nuclear signal but still more than the essentially complete lack of signal in the nuclei of cells infected by the RHΔ*myr1* mutant. Data presented are the pooled data of three independent biological replicates. Error bars indicate standard errors of the means. Values that are significantly different are indicated by a bar and asterisks as follows: **, *P* < 0.0001. (C) Mutations identified by whole-genome sequencing of mutant MFM1.15 and the subclone MFM2.1b. Coverage is the number of reads spanning the indicated nucleotide, and variant frequency is the fraction of reads showing the variant nucleotide relative to the reference (GT1). Both mutants show mutations relative to the annotated type I strain, GT1, in *TGGT1_258580* (*ROP17)* with a missense mutation in MFM1.15 and a nonsense mutation in MFM2.1b.

We previously reported the use of a forward genetic screen to identify *Toxoplasma* genes necessary for the upregulation of mouse c-Myc expression (43). This led to the identification of *MYR1*, *MYR2*, and *MYR3*, and strains with mutations in these genes all show a complete loss of GRA16 or GRA24 translocation across the PVM (43, 44). To determine whether other genes might be involved, we returned to the mutant libraries and screened them for a different phenotype, partial loss of effector translocation. This was done by isolating 42 individual clones from the two libraries and assessing their ability to translocate hemagglutinin (HA)-tagged GRA16 and MYC-tagged GRA24 to the host cell nucleus in infected human foreskin fibroblasts (HFFs). Most of the mutants obtained showed an essentially total loss of such translocation, but one, clone MFM1.15, showed an intermediate phenotype (Fig. 1A and B).

The partial phenotype of MFM1.15 suggested that it might harbor a mutation partially inactivating a gene necessary for translocation, e.g., one of the *MYR* genes, or it might harbor a mutation completely ablating expression of a novel gene that is only partly necessary for translocation and the c-Myc induction. To resolve this, we first used Sanger sequencing to confirm that there was no mutation in the *MYR1*, *MYR2*, or *MYR3* locus and then subjected the clone to whole-genome sequence analysis and identified the 11 mutations shown in Fig. 1C (top panel). None of these mutations were in a known *MYR* gene, but one stood out as being a missense mutation in a gene encoding a known protein kinase present at the PVM, ROP17 (harboring an M350K mutation). This raised the tantalizing possibility that ROP17 might play a role in the translocation of GRA proteins across the PVM. Consistent with this, we returned to previous data sets and saw that a nonsense mutation in *ROP17* had also been seen in one of the original screens that yielded MYR1 (43). In the latter instance, the supposed “clone” that was sequenced, MFM2.1, turned out actually to be a pair of clones, such that all the mutations detected were present in only about 50% of the sequence reads. One of these mutations was in a gene that was mutated in two other (true) clones analyzed in the same set, so this gene was pursued and eventually shown to be essential for c-Myc upregulation and was thus designated *MYR1*. At the time, we did not know which of the other mutations detected were random “hitch-hiker” mutations in the *myr1* mutant versus which might be key to the phenotype in the other clone present in the MFM2.1 pair. To resolve this, we recloned MFM2.1 parasites by limiting dilution and searched for mutants that had a wild-type MYR1 gene. One of these mutants was fully genome sequenced, and the result was mutant MFM2.1b which was found to have the ROP17 S151* mutation, consistent with a defect in ROP17 being the defect that produced the Myr^−^ phenotype (Fig. 1C, bottom panel).

**FIG 2:**
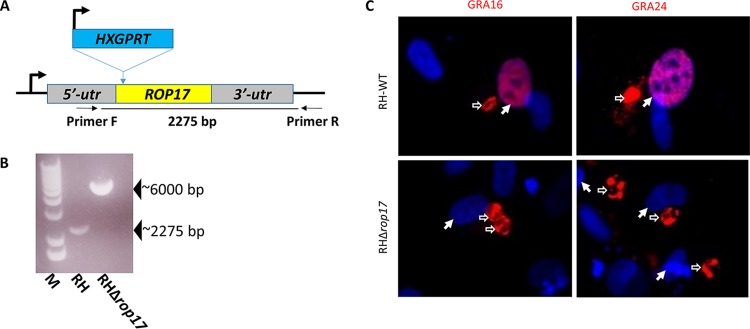
Deletion of *ROP17* generates a mutant parasite that cannot export GRA16 or GRA24 from the parasitophorous vacuole. (A) Strategy for generating a disruption in *ROP17*. Plasmid pTKO2 containing HXGPRT (conferring resistance to mycophenolic acid in an otherwise Δ*hxgprt* strain) was integrated into a cleavage site generated by Cas9 in the beginning of the ROP17 open reading frame. The positions of the primers F and R that were used for detection of the insertion are shown. (B) PCR data showing results of amplification with primers F and R of panel A. The wild-type locus yields a band of ∼2,275 bp, whereas insertion of the knockout plasmid yields a band of ∼6,000 bp. M, molecular markers. (C) IFA of HFFs infected with wild-type RH (RH-WT) or RHΔ*rop17* that had also been transiently transfected with GRA16HA (left) or GRA24MYC (right) and then stained with anti-HA (red, left), or anti-Myc tag (red, right) and DAPI (blue) to reveal the nuclei. Hollow white arrows indicate parasitophorous vacuoles; solid white arrows indicate the nuclei in such cells. Translocation of GRA16 and GRA24 to the host nucleus is seen in cells infected with RH-WT but not RHΔ*rop17* parasites (quantitation of similar experiments is shown in Fig. 5).

The finding that *ROP17* is mutated in both MFM1.15 and MFM2.1b strongly suggested that a functional ROP17 protein might be necessary for the c-Myc upregulation and protein translocation across the PVM. To test this, we generated a knockout of *ROP17* in an otherwise wild-type *Toxoplasma* by disrupting the open reading frame of *ROP17* (*TGGT1_258580*) with insertion of the *HXGPRT* gene (Fig. 2A), confirming this disruption by PCR of the locus (Fig. 2B), and assessing the translocation of known effectors in HFF cells infected with the resulting mutant. Preliminary results indicated that disruption of *ROP17* does indeed prevent the parasite from exporting GRA16HA and GRA24MYC from the parasitophorous vacuole into the host nucleus when these constructs were transiently expressed in RHΔ*rop17* tachyzoites (Fig. 2C). Quantification is presented further below.

**FIG 3:**
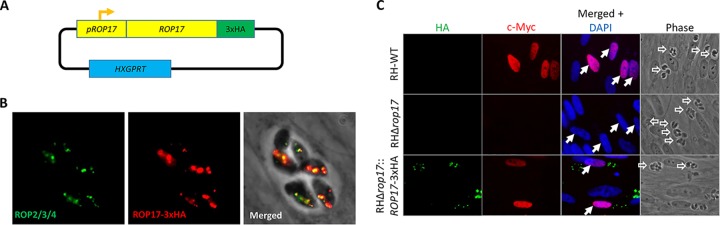
A wild-type copy of *ROP17* rescues the Myr^−^ phenotype of the Δ*rop17* mutant. (A) Strategy for complementing the Δ*rop17* mutants with a 3xHA-tagged wild-type copy of *ROP17*. (B) IFA showing successful complementation of the Δ*rop17* mutant with a HA-tagged wild-type copy of the gene. Green shows staining with anti-ROP2/3/4 as a marker for rhoptries; red shows staining for the complementing ROP17-HA. (C) IFA of HFFs infected with RH-WT, RHΔ*rop17,* or RHΔ*rop17*::*ROP17-3xHA*. Anti-HA antibody was used to detect the complementing ROP17 (green), while red was used for staining of host c-Myc as an indicator of successful effector translocation, and blue shows DAPI staining of the host nuclei. Hollow white arrows indicate parasitophorous vacuoles; solid white arrows indicate the host nuclei in infected cells (quantitation of similar experiments is shown in Fig. 5).

To confirm the importance of ROP17, we generated a complemented Δ*rop17* mutant in which a triple HA-tagged version of ROP17 is expressed off an introduced transgene (Fig. 3A). Within the parasite, ROP17HA colocalized with ROP2/3/4 at the apical end of the parasite (Fig. 3B), supporting proper localization of the tagged protein. As our previous results suggested that ROP17 may be playing a critical role in the export of MYR-dependent proteins, we used the host c-Myc regulation phenotype as a readout of successful complementation. The results (Fig. 3C) showed that cells infected with the Δ*rop17* mutants show only background levels of c-Myc in the nuclei of infected cells, whereas cells infected with the wild type and the complemented mutant show robust c-Myc expression (confirmation and quantification of these results are presented further below). These results thereby confirm that ROP17 is indeed necessary for host c-Myc upregulation by *Toxoplasma* tachyzoites, and in combination with the defect of translocation of GRA16 and GRA24, this strongly suggests that ROP17 is a previously unknown player in the process whereby GRA proteins cross the PVM.

**FIG 4:**
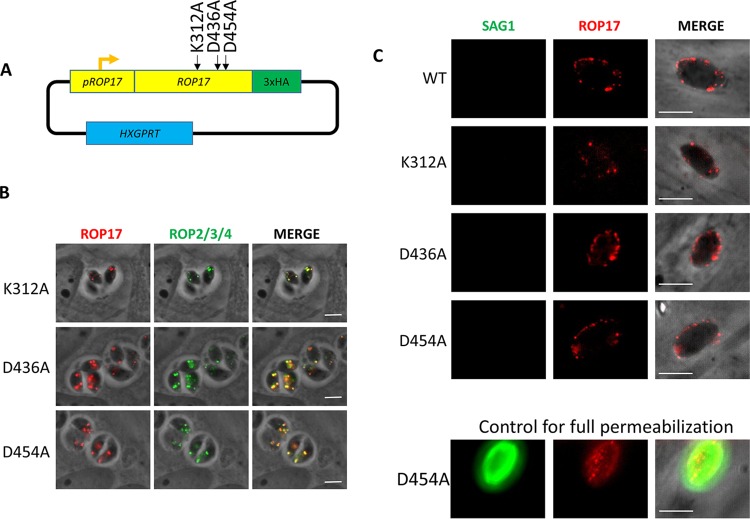
Creation of three strains of RHΔ*rop17* containing point mutations in key catalytic residues. (A) Three different plasmids were created for the expression of point mutant variants of ROP17; each expresses a *ROP17* transgene encoding an alanine substitution at one of the three predicted catalytic residues: K312, D436, and D454. (B) IFA of infected HFFs showing correct trafficking of the mutated ROP17 expressed in an RHΔ*rop17* background. Anti-HA (red) detects the ROP17 transgene product, while anti-ROP2/3/4 (green) detects other known rhoptry proteins. (C) IFA of HFFs that were infected with the strains expressing the indicated HA-tagged version of ROP17 (WT, K312A, D436A, or D454A), partially permeabilized by treatment with 0.02% digitonin, and then stained for ROP17-3xHA using anti-HA (red) or anti-SAG1 (green). The absence of SAG1 staining was used to indicate that the parasitophorous vacuole was not permeabilized, indicating partial permeabilization that allows antibodies to access the host cytosol but not penetrate the PVM. ROP17 is detected at the PVM in cells that are only partially permeabilized, indicating the expected cytosolic exposure of the protein. A positive control of an infected cell that was fully permeabilized under these conditions is shown to confirm that anti-SAG1 staining is readily seen in such cells.

Even though ROP17 is a well-studied serine/threonine protein kinase, it is possible that its role in protein translocation at the PVM is as a scaffolding protein rather than as an active kinase. To test this, we made three different versions of ROP17, each with a mutation to alanine in one of three residues known to be essential for catalysis (45–47), i.e., K312A, D436A, and D454A (Fig. 4A). These mutated versions were introduced into the Δ*rop17* mutant where they showed the expected colocalization with ROP2/3/4 in puncta at the apical end (Fig. 4B). To determine whether the mutant ROP17s reached the PVM after invasion, we applied previously established conditions for partially permeabilizing infected host cells such that antibodies can reach only the PVM, not the parasites within (48). This showed that, indeed, in cells where the control antibody (anti-SAG1) fails to detect the parasites within the PVM, anti-ROP17 efficiently stains the PVM, showing that the mutant ROP17s do successfully enter the host cell and traffic to this location (Fig. 4C).

**FIG 5:**
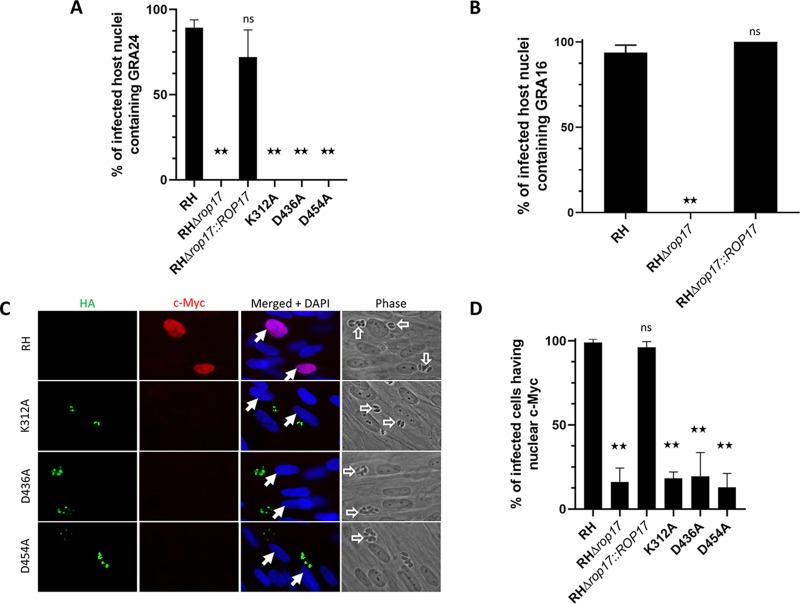
ROP17 catalytic activity appears necessary for its role in effector translocation. (A) HFFs were infected with the indicated strains transiently expressing GRA24MYC, and then 17 to 18 h later, the presence of GRA24MYC in the nuclei of cells infected with GRA24MYC-expressing parasites was assessed by IFA. The results shown are the averages and standard deviations of three technical replicates for each condition and are representative of three biological replicates performed for RH, RHΔ*rop17*, and RHΔ*rop17*::*ROP17* and two biological replicates for the catalytically dead mutants. Differences were assessed by ANOVA with a *post hoc* Tukey’s test. **, *P* < 0.0001; ns, not significantly different. (B) HFFs infected for 18 h with the indicated strains transiently expressing GRA16HA were assessed for the presence of GRA16 in the nucleus. Data shown are from one representative biological replicate of three performed, all of which gave similar results. Differences were assessed by ANOVA with a *post hoc* Tukey’s test. **, *P* < 0.0001. (C) Assessment of the effect of mutating ROP17 on host c-Myc upregulation upon infection. HFFs were infected with the indicated strain, and 20 h later, c-Myc expression in the nuclei of infected cells was assessed using IFA and anti-c-Myc antibodies (red). Expression of the variants of ROP17-3xHA was assessed by staining with anti-HA (green). None of the three catalytic mutants rescued the Myr^−^ phenotype of the Δ*rop17* mutant. (D) Quantitation of *Toxoplasma*’s ability to upregulate host c-Myc was performed by assessing a minimum of 110 random fields from each of three technical replicates for whether an infected cell did or did not express c-Myc. The results shown are from one biological replicate of two performed. Differences between groups were assessed by ANOVA with a *post hoc* Tukey’s test. **, *P* < 0.0001.

We next sought to determine whether the ectopic expression of the wild-type ROP17 gene and/or the mutant versions could complement the phenotypes we have observed in the ROP17-disrupted strains. When we assessed protein translocation of GRA24MYC, we observed translocation into the nuclei of ∼89% of cells infected with GRA24MYC-expressing wild-type parasites, whereas cells infected with GRA24MYC-expressing RHΔ*rop17* parasites showed no such translocation (Fig. 5A). The loss of GRA24 translocation was successfully rescued when the RHΔ*rop17* parasites were complemented with a fully functional ROP17 but not with any of the point mutant versions of ROP17 (Fig. 5A). Similarly, complementation with the wild-type ROP17 returned translocation of the effector protein GRA16 as well (Fig. 5B). When we assessed c-Myc upregulation, a host phenotype associated with the translocation of MYR1-dependent effectors, we observed a similar result; c-Myc was upregulated in >90% of the nuclei of host cells infected with wild-type RH and RHΔ*rop17::ROP17HA* parasites but in <20% of the nuclei of host cells infected with RHΔ*rop17* or any of the three versions complemented with a mutation altering the trio of catalytic residues (Fig. 5C and D). These results strongly suggest that ROP17’s kinase activity is necessary for its role in GRA translocation across the PVM.

**FIG 6:**
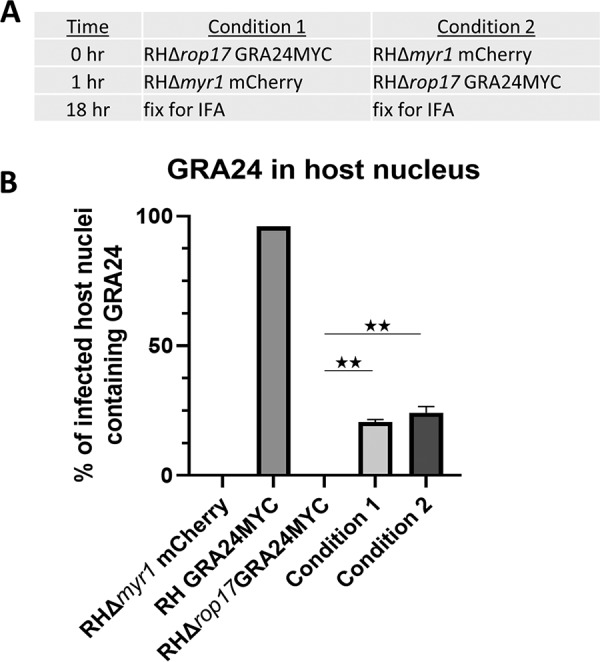
ROP17’s role in effector translocation occurs at the host cytosolic side of the PVM. (A) Conditions used for coinfection of host cells with RHΔ*rop17*::*GRA24MYC* and RHΔ*myr1* expressing mCherry. Infections were initiated with the indicated strain, followed by addition of the second strain 1 h later, followed by IFA after a further 17 h. (B) Quantitation of the percentage of host nuclei staining positive for GRA24MYC in the cells infected with the indicated strains. Cells infected with one or other of the two mutants showed no GRA24MYC, whereas those that were coinfected with both mutants showed substantial rescue in “*trans*.” Data shown are averages of technical replicates and are representative of two independent biological replicates. Differences were assessed by ANOVA with a *post hoc* Tukey’s test. **, *P* < 0.001.

Given that ROP17 ends up at the PVM in infected cells and given that this is where the known GRA translocation machinery (e.g., MYR1/2/3) is located, it seemed most likely that this is where ROP17 functions to assist in the translocation of GRA proteins. To test this directly, we took advantage of the fact that when a tachyzoite infects a cell, the ROP proteins that are injected can associate either with the PVM of that parasite or with the PVM surrounding other parasites that are also present within that cell (49). Thus, we created a reporter parasite line that lacked ROP17 expression but was stably expressing GRA24MYC; translocation of GRA24MYC in this strain should be blocked at the PV unless ROP17 can be provided in *trans*. We then infected cultures with these RHΔ*rop17::GRA24MYC* parasites, followed an hour later with RHΔ*myr1* parasites expressing mCherry to distinguish them from the nonfluorescent RHΔ*rop17::GRA24MYC* line (“Condition 1” in Fig. 6A). In case the order of infection was important, we also did this experiment where we inverted the order that the two strains were added to the monolayers; i.e., we initiated the infections with the RHΔ*myr1* mCherry line, followed an hour later by infection with the nonfluorescent RHΔ*rop17::GRA24MYC* (“Condition 2” in Fig. 6A). In both cases, we assessed GRA24MYC translocation in infected cells after a further 17 h, scoring cells that were infected with either one of the mutants alone or those coinfected with both. The prediction was that if ROP17’s role in GRA translocation is on the host cytosolic side of the PVM, then the Δ*myr1* line would provide a functional ROP17 that could act in *trans* on the translocation machinery expressed by the Δ*rop17* parasites, whereas cells infected with either strain alone would not exhibit translocation. As shown in Fig. 6B, this was indeed the result obtained; cells infected with either mutant alone showed no GRA24MYC in the host nucleus, whereas ∼20 to 22% of coinfected cells did show translocation, regardless of the order that the two strains were added to the cultures. These results strongly argue that the action of ROP17 can be provided in *trans* and is needed within the host cytosol, not within the parasites or within the PV space since proteins are not known to be able to traffic across the PVM from the host to the PV or parasite (except to the lysosome for digestion [50]).

**FIG 7:**
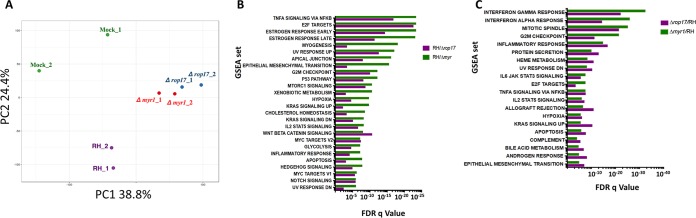
Disrupting *MYR1* or *ROP17* has congruent impacts on the infected host cell’s transcriptome as assessed by RNASeq. (A) Principal-component analysis (PCA) of the RPKM values of HFFs mock infected or infected with RH-WT (wild type), RHΔ*myr1*, or RHΔ*rop17* tachyzoites. On a plot of PC1 and PC2, there is close similarity of the data for cells infected with the two mutants, RHΔ*myr1* and RHΔ*rop17*, relative to the mock- or RH-WT-infected cells. Gene names and RPKMs can be found in Table S1 in the supplemental material. (B) Gene set expression analysis (GSEA) of all genes expressed 2.5-fold higher in RH than in RHΔ*myr1* (green) or RHΔ*rop17* (purple), where either was lower than the FDR q-value threshold of 10^−5^. The gene sets are ordered based on descending levels of significance (ascending q*-*values) for the cells infected with RHΔ*myr1*. (C) As in panel B, except GSEA was performed on the genes that were expressed 2.5-fold lower in RH compared to RHΔ*myr1* and RHΔ*rop17* by the same criteria.

The data presented so far show that ROP17 is necessary for the translocation of at least two GRA proteins from the PV to the host nucleus. We recently reported a detailed analysis of the effects on the host transcriptome that are MYR1 dependent (42). To determine whether infection by parasites lacking ROP17 phenocopies the effect of infection with MYR1-deficient parasites, we performed transcriptome sequencing (RNASeq) on infected HFFs at 6 h postinfection. The results showed substantial concordance between the genes modulated in a MYR1-dependent manner and a ROP17-dependent manner. This conclusion can be illustrated by principal-component analysis (PCA) of the host gene sets generated during infection with these strains. In Fig. 7A, RNASeq data for the eight strains analyzed are shown on the plot of the first and second principal component, and the individual genes along with their RPKM values are displayed in Table S1 in the supplemental material. The samples infected with RHΔ*myr*1 and RHΔ*rop17* cluster together closely and well apart from both mock-infected cells and RH-infected cells.

10.1128/mSphere.00276-19.1 TABLE S1 List of the RPKM values of HFFs infected with the respective strains. Download Table S1, XLSX file, 2.2 MB. Copyright © 2019 Panas et al. 2019 Panas et al. This content is distributed under the terms of the Creative Commons Attribution 4.0 International license.

To further explore this similarity, genes that exhibit a 2.5-fold difference during infection with these mutants compared to infection with the wild type were grouped by gene set enrichment analysis (GSEA). First, genes that were increased in cells infected with the wild-type parasite (RH) compared to the two mutants were analyzed by GSEA. Figure 7B shows a list of gene sets in which the false-discovery rate (FDR) q-value of either the RH versus RH*Δmyr1* or the RH versus RH*Δrop17* was less than 10^−5^. For each of these gene sets, both the FDR q-value of the RH versus RH*Δmyr1* (green) and RH versus RH*Δrop17* (purple) is shown. The most significantly enriched pathway, TNFA signaling via NF-κB, is dependent upon both MYR1 and ROP17. Similarly, E2F targets, the second-most-enriched gene set, is dependent upon both MYR1 and ROP17. Our and others’ recent findings that HCE1/TEEGR is necessary and sufficient for the upregulation of both E2F targets and the G_2_M checkpoint in HFFs would suggest that this effector protein is among the many MYR1-dependent proteins that are also ROP17 dependent (51, 52).

Genes that were expressed 2.5-fold lower in cells infected with the wild type compared to either of the mutants were also analyzed by GSEA and gene sets in which either the FDR q-value of RH versus RH*Δmyr1* (green) or RH versus RH*Δrop17* was less than 10^−5^ were plotted in Fig. 7C. We observed some enrichment of gene sets that were redundant to the enrichment observed in upregulated genes. For example, “G_2_M checkpoints” was enriched in both the upregulated genes and downregulated genes. This occurs because entire pathways are being perturbed by *Toxoplasma*’s effector proteins, and within these pathways, simultaneous upregulation of some genes and downregulation of other genes are occurring.

In both cases, almost all the gene sets that are strongly affected by the lack of *MYR1* were similarly affected by the lack of *ROP17*, although the magnitude of the effect varied somewhat. Finally, we performed a direct comparison of expression levels in cells infected with RHΔ*myr1* and cells infected with RHΔ*rop17* (instead of comparing each to the wild-type-infected cells). We used GSEA to analyze genes for which expression was 2.5-fold higher or 2.5-fold lower in RHΔ*rop17*-infected cells than in RHΔ*myr1*-infected cells. The results were that in neither case was a gene set enriched with an FDR q-value of even a very relaxed threshold of 10^−4^. Hence, the absence of ROP17 and MYR1 appear to have similar impacts on the infected cell, and thus, ROP17 appears necessary for the action of most, probably all, GRA effectors that transit across the PVM via the MYR machinery.

## DISCUSSION

Using a genetic screen, we have identified the rhoptry-derived serine-threonine protein kinase, ROP17, as required for action of most, if not all, GRA effectors that translocate across the PVM. Using a cellular “trans” complementation assay, we have further shown that the role of ROP17 is within the host cytosol, not within the parasite or PV space, and that ROP17 must be catalytically active to accomplish this role. Given its location at the PVM (13, 21), where other necessary elements of the translocation machinery are present, these results strongly argue that ROP17 acts on one or other components of this machinery on the host cytosol side. Although we cannot formally exclude the possibility that ROP17 assists GRA effectors in their trafficking across the host cytosol, from the PVM to the host nucleus, the fact that the GRA effectors that reach the host nucleus possess a conventional nuclear localization signal (NLS) argues against this possibility, as there should be no need for any additional help in their journey. Indeed, heterologous expression of GRA16 and GRA24 in uninfected cells shows results in efficient trafficking to the host nucleus, confirming that no parasite proteins are necessary for this last stage of their journey (37, 38).

Given that ROP17 is a protein kinase, it seems most likely that its role in translocating GRA effectors is through phosphorylation of one or more key components of the translocation machinery. Phosphoproteomic analyses on cells infected with *Toxoplasma* tachyzoites revealed that many parasite proteins are phosphorylated at serine and threonine residues after their secretion from the parasite (53). Among such proteins are the PVM-localized MYR1 and MYR3 that are known to be required for GRA translocation (43, 44). The protein kinases that mediate these phosphorylations have not yet been identified, but protein phosphorylation is a well-established way to regulate protein function, so such modifications might be required for PVM-localized proteins like these to become activated for their respective roles. Efforts to determine the full machinery involved in GRA translocation across the PVM are under way, and once the full complement of proteins is known, mapping of all their phosphosites and determination of which such sites are dependent on which protein kinase (e.g., ROP17 or, perhaps, ROP18, another serine-threonine kinase present at the PVM) and which of these sites must be phosphorylated for functional translocation will be an important follow-up to the work proposed here.

Both ROP17 and ROP18 are involved in the inactivation of immunity-related GTPases (15, 16, 21). Our finding that ROP17 has at least two biological roles is similar to what has been reported for ROP18; this related ROP2 family member is involved in IRG inactivation and proteasomal degradation of ATF6beta, a host transcription factor that localizes to the host endoplasmic reticulum (which itself is adjacent to and maybe even contiguous with the PVM [54]) and that is crucial to the host immune response (55). Interestingly, it is the N-terminal region of ROP18, which lies outside the conserved kinase domain, that binds to ATF6beta, but an active kinase domain is required for the inactivation, suggesting that ATF6beta is a substrate for phosphorylation by ROP18 (55). Our results add a further possible explanation for the previously reported attenuation of ROP17 mutants in a mouse model of virulence using a type I strain (21); i.e., the decrease in virulence could be due to some combination of a weakened defense against IRGs and the defect in GRA effector translocation reported here. A major role for the latter would be consistent with the previously reported attenuation in type I Δ*myr1* strains in a similar mouse model (43).

The involvement of a rhoptry protein in the function of GRA proteins is a second example of “interorganellar” collaboration, the first being the binding of micronemal AMA1 to rhoptry neck protein RON2 during the invasion of tachyzoites into the host cell (56, 57). This second example wherein a rhoptry bulb protein, ROP17, somehow assists in the translocation of GRA proteins makes clear that these different secretory organelles are part of a complicated but concerted machinery used by the parasites to interact with the host cell they are infecting.

Finally, it is worth noting that the chemical mutagenesis used to generate the mutant library that yielded these *ROP17* mutants provided more information than just the fact that this protein plays an important role. By specifically looking for hypomorph mutants that showed only a partial defect, we were able to identify a missense M350K mutation in ROP17 providing structure/function information, namely, that this residue is important for this function of ROP17. This site is within a predicted loop region that is just N terminal of beta sheet 4 and well away from the active site (13, 45, 46). Interestingly, this is part of a region that is highly variable between different members of the ROP2 family but is in a stretch of about nine residues that is not present in other protein kinases (46). This may be related to the unusual, multiple functions of these secreted kinases, perhaps in enabling them to target specific substrates at this crucial interface of host and parasite.

## MATERIALS AND METHODS

### Parasite culture.

Toxoplasma gondii RHΔ*hpt* (58) was used for this study. *Toxoplasma* tachyzoites were maintained by serial passage in human foreskin fibroblasts (HFFs) cultured in complete Dulbecco’s modified Eagle medium (cDMEM) supplemented with 10% heat-inactivated fetal bovine serum (FBS), 2 mM l-glutamine, 100 U/ml penicillin, and 100 μg/ml streptomycin and grown at 37°C in 5% CO_2_. Infections included in this study were performed by scraping infected monolayers and lysing the host cells open using a 27-gauge needle. The released parasites were pelleted at 1,500 rpm for 10 min, resuspended, counted using a hemocytometer, and added to confluent HFFs at the multiplicity of infection (MOI) stated.

### Genome sequencing.

For whole-genome sequencing on the parental RH strain (SRR2068658), MFM1.15 (SRR5643318), and MFM2.1b (SRS2249312) mutants, a single Illumina PE barcoded library was prepared from tachyzoite gDNA. Libraries were then pooled into groups of nine samples and multiplex sequenced in a single lane of an Illumina HiSeq 2000 machine to generate about 3 Gb of sequencing data per sample. As the mutants were made using the type I RH strain as the parent, the sequencing reads were first quality trimmed with trimommatic and then mapped to the reference assembly of the type I GT1 strain (as present in ToxoDB v13.0) with *bowtie2* (59). After removing duplicated reads with *picard* and adjusting alignments around indels with *GATK toolkit* (60), single nucleotide variants (SNVs) were called using samtools utility *mpileup* (61) requiring a minimum base coverage of five reads and an alternative allele frequency of at least 80% or higher. Following this, *SnpEff* (62) together with a gff3 annotation file from the reference GT1 strain (ToxoDB v13.0) were used to classify the different types of SNVs present in each mutant. Potential change-of-function SNVs that were different in any of the two mutants and both the parental and reference strains were selected for further quality control and analysis.

### Transfections.

All transfections were performed using the *BTX* EMC600 Electroporation System (Harvard Apparatus) or Amaxa 4D Nucleofector (Lonza) model. Tachyzoites were mechanically released in phosphate-buffered saline (PBS), pelleted, and resuspended in solution for transfection. After transfection, parasites were allowed to infect HFFs in DMEM. Transfections with the *BTX* EMC600 model were performed using 5 × 10^6^ to 10 × 10^6^ parasites and 5 to 10 μg DNA in Cytomix (10 mM KPO_4_ [pH 7.6], 120 mM KCl, 5 mM MgCl_2_, 25 mM HEPES, 2 mM EDTA, 150 μM CaCl_2_). Transfections with the Amaxa 4D model were performed using 1 × 10^6^ to 2 × 10^6^ parasites in 20 μl of P3 solution or 5 × 10^6^ to 10 × 10^6^ parasites in 100 μl of P3 solution with 5 to 15 μg DNA. Effector translocation assays were performed by transiently transfecting pHTU-GRA24MYC (44) or pGRA1-GRA16HA (44) plasmid into tachyzoites, infecting monolayers of HFFs in DMEM, and fixing monolayers with formaldehyde at 16 to 24 h postinfection (hpi).

### Immunofluorescence microscopy.

Infected cells grown on glass coverslips were fixed using methanol at –20°C for 20 min or 4% formaldehyde at room temperature (RT) for 20 min, as stated in the text. Methanol-fixed samples were washed three times for 5 min with PBS and blocked using 3% bovine serum albumin (BSA) in PBS for 1 h at RT. Formaldehyde-fixed samples were rinsed once with PBS, permeabilized with 0.2% Triton X-100 (TTX-100) for 20 min and then blocked as described above. GRA16HA (and other hemagglutinin [HA]-tagged proteins) was detected using rat anti-HA antibodies (Roche), while GRA24MYC was detected using rabbit anti-MYC tag antibody 9E10 (Santa Cruz Biotechnology). This anti-MYC tag antibody does not detect host c-Myc. Host c-Myc was detected using monoclonal antibody Y69, which does not cross-react with the MYC tag expressed by GRA24MYC. Primary antibodies were detected with goat polyclonal Alexa Fluor-conjugated secondary antibodies (Invitrogen). Vectashield with 4′,6′-diamidino-2-phenylindole (DAPI) stain (Vector Laboratories) was used to mount the coverslips on slides. Fluorescence was detected using a LSM710 inverted confocal microscope (Zeiss) or epifluorescence microscope as stated in the text. Images were analyzed using ImageJ. All images shown for any given condition/staining in any given comparison/data set were obtained using identical parameters.

### Quantitation of nuclear GRA16HA.

To quantify the amount of GRA16HA that translocated to the nucleus following transient transfection, phase-contrast, DAPI, and anti-HA tag images were taken of 10 to 20 fields of view containing tachyzoite-infected HFFs at 20 h postinfection (p.i.). Phase-contrast microscopy was used to define the infected cells of these images, and then ImageJ was used to define the nucleus on the DAPI-stained corresponding images, and these nuclear boundaries were then quantified for the intensity of GRA16HA on the corresponding HA tag-stained images.

### Partial permeabilization.

Parasites were syringe released using a 27-gauge needle and used to infect HFFs for 2 h, at which time the cells were washed with PBS and then fixed with 4% formaldehyde at room temperature for 20 min. Formaldehyde-fixed samples were rinsed once with PBS, permeabilized with 0.02% digitonin solution for 5 min, and then blocked with 3% BSA in PBS for 1 h at RT. Staining was performed with anti-HA (Roche) and anti-SAG1 (DG52) primary antibodies and polyclonal Alexa Fluor-conjugated secondary antibodies (Invitrogen). Partial permeabilization of a particular vacuole was determined by the exclusion of the SAG1 antibody.

### Gene disruption.

The RHΔ*rop17* strain was generated by disrupting the corresponding gene locus using CRISPR-Cas9 and selecting for integration of a linearized vector encoding hypoxanthine-guanine phosphoribosyl transferase (*HXGPRT*) using drug selection for 8 days using 50 μg/ml mycophenolic acid (MPA) and 50 μg/ml xanthine (XAN) for HXGPRT selection. Specifically, the pSAG1:U6-Cas9:sgUPRT vector (63) was modified by Q5 site-directed mutagenesis (New England BioLabs [NEB]) to specify sgRNAs targeting *ROP17* (F2). The resulting sgRNA plasmid, dubbed pSAG1:U6-Cas9:sgROP17 (P1) was transfected into the RHΔ*hpt* strain of *Toxoplasma* with pTKO2 (HXGPRT+) plasmid. The parasites were allowed to infect HFFs in 24-well plates for 24 h, after which the medium was changed to complete DMEM supplemented with 50 μg/ml mycophenolic acid and 50 μg/ml xanthine for HXGPRT selection. The parasites were passaged twice before being singly cloned into 96-well plates by limiting dilutions. Disruption of the gene coding regions was confirmed by PCR and sequencing of the locus.

### Ectopic gene integration.

The RHΔ*rop17* strain was complemented ectopically with the pGRA-ROP17-3xHA plasmid, which expresses *ROP17* off its natural promoter. To construct the pGRA-ROP17-3xHA plasmid, pGRA1_plus_-HPT-3xHA plasmid (44) was first digested using EcoRV-HF and NcoI (New England Biolabs) for 4 h at 37°C to remove the *GRA1* promoter. Product was incubated with Antarctic phosphatase (New England Biolabs) and gel extracted. The empty vector backbone was amplified by PCR using Herculase II polymerase (Agilent) and primers 5′-CACATTTGTGTCACCCCAAATGAGAATTCGATATCAAGCTTGATCAGCAC-3′ and 5′-GAGGCGGCTTTATTACAGAAGGAGCCATGGTACCCGTACGACGTCCCG-3′ with each having 23- and 24-bp overhangs to *ROP17*, respectively. The *ROP17* promoter and open reading frame were amplified from RHΔ*hpt* genomic DNA and using 5′-GTGCTGATCAAGCTTGATATCGAATTCTCATTTGGGGTGACACAAATGTG-3′ and 5′-CGGGACGTCGTACGGGTACCATGGCTCCTTCTGTAATAAAGCCGCCTC-3′ primers, each containing 27- or 24-bp overhangs to the pGRA1_plus_-HPT-3xHA plasmid backbone, respectively. Amplified backbone and *ROP17* were then assembled using the Gibson assembly master mix (New England Biolabs). ElectroMAX DH10B Escherichia coli (Invitrogen) bacteria were subsequently transformed and plated to obtain single colonies of successfully assembled pGRA1-ROP17-3xHA plasmid. *ROP*17 integration was verified by PCR and sequencing using primers 5′-CACTGATCGGCTTTGTAGACTT-3′ and 5′-CGCGCACGGCAGTCAGATAA-3′.

To complement RHΔ*rop17*Δ*hpt* parasites with wild-type *ROP17*, the pGRA1-ROP17-3xHA plasmid construct described previously was transfected to generate an RHΔ*rop17*::*ROP17* population. This population was selected by MPA/XAN as previously described. The resulting population was then cloned by limiting dilution and tested for ROP17-3xHA expression by Western blotting and immunofluorescence assay (IFA).

To generate RH and RHΔ*rop17* parasite lines ectopically expressing GRA24MYC, pHTU-GRA24-3xMYC was transfected into each strain and selected using MPA/XAN as described above for 6 days.

### Site-specific mutation.

Site-specific point mutation of the pGRA-ROP17-3xHA plasmid was performed by creating primers 5′-GCGATATTTGTTCAACGGGTGTTGAGCAAT-3′ and 5′-CAGCGCGAATGGTTGCCCTGTGGTGGG-3′, 5′-GCTGTGAAACTGCAAAATTTTCTTGTTGAT-3′ and 5′-GCCATGAACAAGTCCGAACGCGTGGAA-3′, and 5′-GcCTTCACTCAAATTCTTCGTACGAATG-3′ and 5′-AGAAAGTAGAAGCAATCCCGATTTATC-3′ to mutate residues 312, 436, and 454, respectively, to an alanine codon within the *ROP17* open reading frame. The “Round-the-horn” site-directed mutagenesis approach was used to introduce point mutations at the aforementioned residues using the ROP17-3xHA plasmid. The PCR products were then individually ligated using a KLD Enzyme reaction kit (New England Biolabs) for 3 h and subsequently transformed into ElectroMAX DH10B E. coli (Invitrogen). Single colonies for each point mutant were miniprepped (Qiagen) and sequence verified using either 5′-GCCATGAACAAGTCCGAACGCGTGGAA-3′ or 5′-GCGATATTTGTTCAACGGGTGTTGAGCAAT-3′.

To generate parasite lines complemented with the catalytically inactive *ROP17*, RHΔ*rop17* parasites were transfected with the pGRA1-ROP17_K312A_-3xHA, pGRA1-ROP 17_D436A_-3xHA, or pGRA1-ROP17_D454A_-3xHA plasmid and then subsequently selected for 6 days with MPA/XAN and singly cloned as previously described.

### Coinfection assays.

In condition 1, confluent HFF coverslips were infected with RHΔ*rop17* parasites stably expressing GRA24-3xMYC at an MOI of 0.15. They were then pulsed at 1,400 rpm and placed at 37°C and 5% CO2 for 1 h. Thereafter, the same sample was infected with RHΔ*myr1* constitutively expressing mCherry at an MOI of 0.15, pulsed at 1,400 rpm, and placed at 37°C and 5% CO_2_. Infections were allowed to progress for 17 to 18 h. Condition 2 was performed in the same way except the order of addition of the two strains to the host cells was reversed.

### RNA extraction, library preparation, and sequencing.

HFFs were serum starved for 24 h before infection by growth in DMEM containing 0.5% serum. They were then infected with the indicated line of tachyzoites at an MOI of 5, and at 6 hpi, 1 ml of TRIzol reagent (Invitrogen) was added to each T25 and the cells were scraped. Lysates were collected and frozen at −20°C. Total RNA was extracted following the manufacturer’s instructions with some modifications. Briefly, frozen samples were thawed on ice, and 0.2 ml chloroform was added to TRIzol suspensions, which were then mixed by inverting 10 times, and incubated for 5 min. Tubes were then spun at 12,000 rpm for 15 min at 4°C. RNA in the aqueous phase was transferred into a fresh tube, and 0.5 ml absolute isopropyl alcohol was added and incubated at 4°C for 10 min. They were then spun at 12,000 rpm for 20 min at 4°C. After decanting the supernatants, RNA pellets were washed with 1 ml of 75% ethanol and then spun at 12,000 rpm for 20 min at 4°C. Supernatants were removed, and the RNA pellets were resuspended in 30 μl RNase-free diethyl pyrocarbonate (DEPC)-water. RNA samples were submitted to the Stanford University Functional Genomic Facility (SFGF) for purity analysis using the Agilent 2100 Bioanalyzer. Multiplex sequencing libraries were generated with RNA Sample Prep kit (Illumina) according to the manufacturer’s instructions and pooled for a single high-throughput sequencing run using the Illumina NextSeq platform (Illumina Nextseq 500 model instrument).

### RNASeq read mapping and differential expression analysis.

Raw reads were uploaded onto the CLC Genomics Workbench 8.0 (Qiagen) platform for independent alignments against the human genomes (Ensembl.org/hg19) and *Toxoplasma* type I GT1 strain (ToxoDB-24, GT1 genome). All parameters were left at their default values. The number of total reads mapped to each genome was used to determine the RPKM (reads per kilobase of transcript per million mapped reads). Among these genes, only those with an average RPKM ratio of ≥2.5 were counted as showing changed expression. Equivalent MOI of each sample was confirmed by approximately equivalent reads mapping to *Toxoplasma* versus the host (i.e., 48% and 61% in the two RH wild-type [WT] samples, 51% and 49% in the two RHΔ*myr1* samples, and 58% and 69% in the two RHΔ*rop17* samples).

### Gene set enrichment analysis.

Gene set enrichment analysis (GSEA), available through the Broad Institute at http://www.broadinstitute.org/gsea/index.jsp, was the enrichment analysis software we used to determine whether defined sets of differentially expressed human genes in our experiment show statistically significant overlap with gene sets in the curated Molecular Signatures Databases (MsigDB) Hallmark gene set collection. We used the false-discovery rate (FDR) q-value cutoff of <10^−5^.

### PCA analysis.

To generate PCA (principal-component analysis) plots, we used https://biit.cs.ut.ee/clustvis/#tab-9298-7 online tool. We used the RPKM values of all expressed genes to generate the PCA plots.

### Statistical analyses.

Statistical analysis was performed with Prism version 8 software. For intensity analysis, GRA16HA translocation was assessed by ImageJ, and then differences in intensity were analyzed by one-way analysis of variance (ANOVA) with a *post hoc* Tukey’s test. Similarly, differences in the number of infected host cells with nucleus staining positive for GRA24 translocation or c-Myc expression were compared using a one-way ANOVA with a *post hoc* Tukey’s test.

### Data availability.

The RNASeq data files have been deposited in GEO under accession number GSE133144. Data presented as transcriptomic control data, for uninfected HFFs and HFFs infected by RH and RHΔ*myr1* tachyzoites, have been published under accession number GSE122786 (52).
